# Female alcohol consumption and fecundability: a systematic review and dose-response meta-analysis

**DOI:** 10.1038/s41598-017-14261-8

**Published:** 2017-10-23

**Authors:** Dazhi Fan, Li Liu, Qing Xia, Wen Wang, Shuzhen Wu, Guo Tian, Ying Liu, Jing Ni, Song Wu, Xiaoling Guo, Zhengping Liu

**Affiliations:** 1 Foshan Institute of Fetal Medicine, Southern Medical University Affiliated Maternal & Child Health Hospital of Foshan, Foshan, Guangdong, 528000 China; 2 Department of Obstetrics, Southern Medical University Affiliated Maternal & Child Health Hospital of Foshan, Foshan, Guangdong, 528000 China; 3 Department of Epidemiology and Biostatistics, School of Public Health, Anhui Medical University, Hefei, Anhui 230032 China; 4 Department of Library, the First Affiliated Hospital, College of Medicine, Zhejiang University, Hangzhou, Zhejiang, 310003 China; 5 Department of Maternal, Child and Adolescent Health, School of Public Health, Anhui Medical University, Hefei, Anhui 230032 China; 6 School of Integrated Traditional and Western Medicine, Anhui University of Chinese Medicine, Hefei, Anhui 230038 China

## Abstract

To what extent could alcohol consumption affects female fertility is still unclear. The aim of this study was to quantitatively summarize the dose-response relation between total and specific types of alcohol beverage (beer, wine, and spirits) consumption in female and the fecundability. Four electronic databases were searched. Observational studies (cohort and case-control) that provided female alcohol consumption and fecundity were eligible. Nineteen studies, involving 98657 women, were included in this study. Compared to non-drinkers, the combined estimate (with relative risk, RR) of alcohol consumers on fecundability was 0.87 (95% CI 0.78–0.95) for overall 19 studies. Compared to non-drinkers, the pooled estimates were 0.89 (95% CI 0.82–0.97) for light drinkers (≤12.5 g/day of ethanol) and 0.77 (95% CI 0.61–0.94) for moderate-heavy drinkers (>12.5 g/day of ethanol). Moreover, compared to non-drinkers, the corresponding estimates on fecundability were 0.98 (95% CI 0.85–1.11), 1.02 (95% CI 0.99–1.05), and 0.92 (95% CI 0.83–1.01) for studies focused on wine, beer and spirits, respectively. Dose-response meta-analysis suggested a linear association between decreased fecundability and every 12.5 g/d increasing in alcohol consumption with a RR 0.98 (95% CI 0.97–0.99). This first systematic review and meta-analysis suggested that female alcohol consumption was associated with a reduced fecundability.

## Introduction

Infertility, defined as the inability to conceive after 12 months of unprotected intercourse, is growing a major public health issue^1^. There are approximately 48.5 million infertile couples worldwide^2^ and the prevalence of infertile was estimated between 12.5% and 24% among all couples^3,4^. Many well-defined risk factors, such as diminished ovarian reserve, endometriosis and tubo-peritoneal factors, have been confirmed to be associated with infertility. However, there remain some risk factors on infertility were not fully understood. Modifiable lifestyle risk factors, such as obesity, exercise, diet, smoking, caffeine use, and alcoholic beverage drinking, have been proposed and investigated thoroughly.

Among these lifestyle factors, many observational studies have been published on the topic of alcohol consumption in women and its effects on the development of fecundability. However, whether alcohol consumption could influence fecundability remains unclear and even controversial. Some studies have concluded that low to moderate levels of alcohol consumption is associated with decreased fertility^5–7^. By contrast, data from other observational studies^8–13^ have indicated there is no or even a positive association between moderate alcohol consumption and fertility in women and men^14,15^. For beverage-specific effects on fertility, a large birth cohort study reported that women who drink wine need less time to get pregnancy than women who did not^9^. Inconsistent results between previous studies might attribute to the differences in study design, adjustment of possible confounding factors, and assessment methods of alcohol consumption.

Previous systematic review indicated that heavy alcohol consumption during pregnancy increased the risk of low birth weight and preterm birth^16^. No alcohol intake was recommended for women who are in or preparing for pregnancy, as well as for lactating women; meanwhile, a maximum weekly alcohol intake was also recommended for general healthy women^17^. While it is important to clarify the association between female alcohol consumption and fecundability, there is currently, no study has, in a dose-response fashion, quantificationally calculated the least requirement of reducing alcohol consumption to lower the risk of fecundity using all available data sources. Accordingly, by using a systematic review and meta-analysis, the aim of this study was to summarize available evidence on female alcohol consumption, including overall and specific types of alcoholic beverage (beer, wine, and spirits) consumption, and the risk of fecundability.

## Results

### Study characteristics and quality assessment

**Figure 1:**
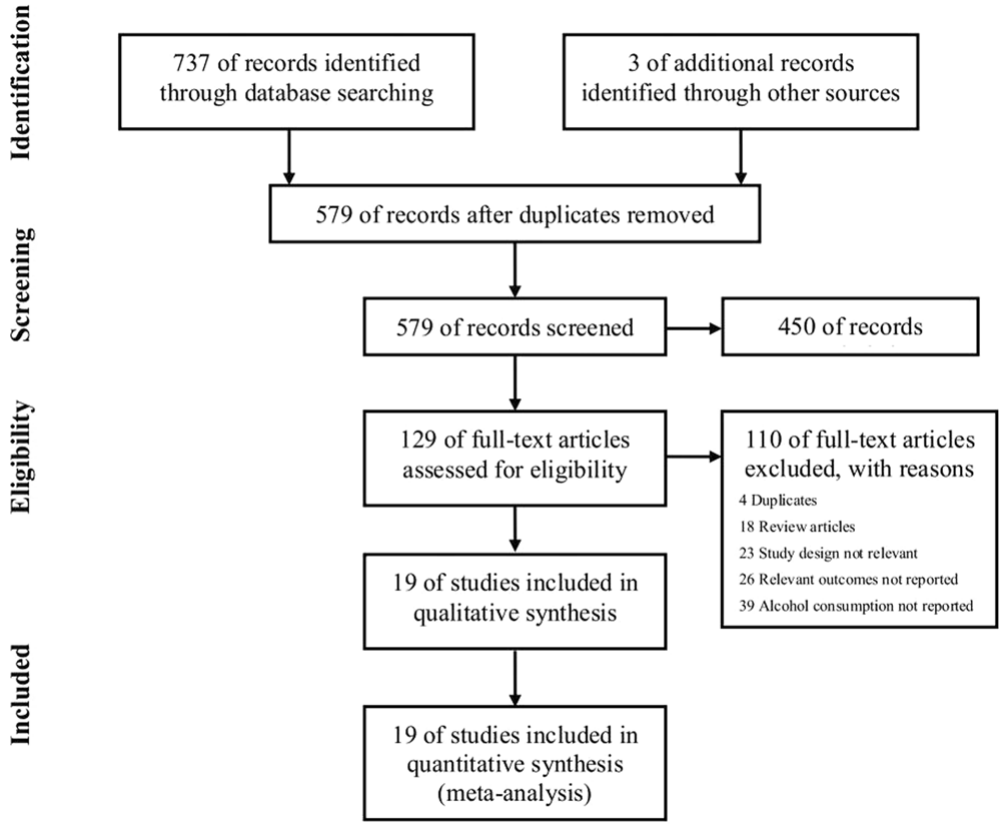
Flow chart for search and selection of studies for inclusion in this meta-analysis.

The literature search strategy identified a total of 740 potentially eligible studies. After removal of the duplicate citations, 579 studies remained for title and abstract screening, of which 129 studies were potentially relevant for full text review. We excluded 110 articles due to duplication and no sufficient information. Finally, nineteen unique studies, including 12 cohort studies^6,8,10,11,14,15,18–23^ and 7 case-control studies^5,7,9,24–27^, met all of the inclusion criteria and were included in the meta-analysis (Fig. 1).

The detailed characteristics of the eligible studies were summarized in Table 1. A total of 98657 women of reproductive age were observed among these studies which were published between 1984 and 2016. Six studies were conducted in the USA^6,7,19,22,23,25^, four in Denmark^5,9,18,21^, two in Canada^11,26^, two in the Netherlands^14,15^, and one for each in Italy^27^, Spain^24^, UK^8^, Sweden^20^ and European multicenter^10^, respectively. Eight studies selected participants from general population^5,9,10,18,20,21,23,24^, five studies from hospital clinics^7,8,14,22,27^; three studies choosed participants from workers^6,15,25^, two studies from agriculturist women^11,26^, and one studies choosed nurses as the participants^19^. Women’s mean age was under 25 years in two studies^20,21^, 25–30 years in ten studies^5,7,8,10,11,14,15,18,24,26^, more than 30 years old in five studies^6,9,19,25,27^, and two studies^22,23^ did not report the women’s age. The time between alcohol consumption and outcome was two years in six studies^6,14,19,24,26,27^, one year in nine studies^7–10,15,18,20,21,23^, and half-year in four studies^5,11,22,25^.

**Table 1:** Characteristics of the studies included in the meta-analysis.

First author, year	Country/Population	Study design	Period of enrolment	Total number	Age in years (Range or SD)	Time exposure	Exposure assessment/ Alcohol unit	Alcohol consumption group	Outcome defined	Adjusted confounding factors	NOS score
Mikkelsen (2016)	Denmark/ Danish residents	Cohort	2007–2016	4210	28 (21–45)	One year	SAQ/ Servings per week	Any alcohol: None; 1–3 servings/week; 4–7 servings/week; 8–13 servings/week; ≥14 servings/week; Wine/Beer/Spirits Only: None; 1 servings/week; 2 servings/week; ≥ 3 servings/week;	Waiting time to pregnancy (number of menstrual cycles < 12 menstrual cycles)	Woman’s and male partner’s age at baseline, vocational training, cycle regularity, parity, current smoking, intercourse frequency, timing of intercourse, body mass index, physical activity, sexually transmitted diseases, caffeine intake, and last method of contraception	8
Chavarro (2009)	USA/Nurses	Cohort	1991–1999	26533	32.6 (24–42)	Two years	FFQ/Drinks per day	Any alcohol: None; 0.1–0.9g/d; 2–4.9g/d; 5–9.9g/d; ≥10g/d; Wine/Beer/Spirits only: None; < 1/mo; 1–3/mo; 1/wk; ≥2/wk;	Self-reported diagnosis of ovulatory disorder infertility	Age, calendar time, total energy intake, BMI, parity, smoking history, physical activity, oral contraceptive use, dietary quality score and caffeine intake	8
Hassan (2004)	UK/Consecutive women attending the antenatal clinics	Cohort	2000–2001	2112	27.4	One year	SAQ/Unit per week	Any alcohol: None; ≤ 20 unit/wk; > 20unit/wk;	Waiting time to pregnancy < 12 months	Women’s age, weight, smoking, tea/coffee intake, drug abuse, parity, contraceptive use, and menstrual pattern, and men’s age, smoking, alcohol consumption, drug abuse, coital frequency, and living standard	8
Eggert (2004)	Sweden/ Random women inhabitants of Stockholm County, Sweden	Cohort	1969–1987	7393	22.21(18–28)	One year	SAQ/Drink per week	Any alcohol: ≤50g/week; 50–140g/week; ≥140g/week;	Diagnoses in accordance with the Manual of International Classification of Diseases, Injuries and Causes of Death 1967 (ICD-8) and 1977 (ICD-9)	Women’s age	7
Tolstrup (2003)	Denmark/ Randomly sampled from the general population in Denmark	Cohort	1991–1993	7760	20–29	One year	FFQ/Drink per week	Any alcohol: <1 per week; 1–6 per week; 7–13 per week; ≥14 per week;	Diagnoses in accordance with ICD-8 and ICD-10	Smoking, pelvic inflammatory disease, marital status and school education	8
Hakim (1998)	USA/Healthy volunteers in manufacturing facilities	Cohort	1989–1991	124	31 (23–41)	Two years	SAQ/Drink per week	Any alcohol: None; 1–12 g/wk; 13–19 g/wk; ≥ 91 g/wk;	Obtaining a clinically recognized pregnancy (hCG)	Age, race, education, pregnancy and fertility history, number of days of intercourse during a cycle, and smoking	7
Olsen (1997)	Denmark; Germany; Italy; Poland; Spain; Sweden;/ Random samples of women in Europe	Cohort	1991–1993	4000	26.3 (4.4)	One year	SAQ/Drink per week	Any alcohol: None; 1–7 drinks/week; 8–21 drinks/week; ≥ 22 drinks/week;	Waiting time to pregnancy > 12 months	Mothers’ education, job (employment and working hours), age, parity, alcohol, and coffee consumption, use of oral contraceptives within 12 months before the starting time, and frequency of sexual intercourse	8
Curtis (1997)	Canada/ Agricultural women	Cohort	1991–1992	2607	25.62 (17–33)	Six months	SAQ/Drink per week	Any alcohol: None; 0.1–1 ounces/week; 1.1–2 ounces/week; > 2 ounces/week;	Occurrence of a pregnancy	Spouse's alcohol use, woman's age when beginning to try to conceive, recent oral contraceptive use, and men's and women's smoking	8
Zaadstra (1994)	Netherlands/Normal healthy women enrolled in a clinic	Cohort	1986–1988	489	29.1 (4.4)	Two years	SAQ/Glasses per week	Any alcohol: None; < 10 glasses per week; ≥ 10 glasses per week;	Pregnancy referred to the clinic by a gynecologist	Smoking, coffee consumption, age, body fat distribition (waist-to-hip ratio), BMI, socioeconomic status, duration of menstrual cycle, and parity	6
Florack (1994)	Netherlands/Nonmedical hospital workers	Cohort	1987–1989	259	18–39	One year	SAQ/Drinks per week	Any alcohol: < 5 drinks per week; 5–10 drinks per week; ≥ 10 drinks per week;	The probability of becoming pregnant each month and was estimated by the time to pregnancy	Age, previous number of pregnancies (gravidity), previous spontaneous abortions, medical drug utilization, current chronic disease, and educational level	8
Joesoef (1993)	USA/Women from clinics	Cohort	1981–1983	2817	NA	Five months	SAQ/Glasses per week	Any alcohol: None; 1–2 drinks/wk; 3–5 drinks/wk; > 5 drinks/wk;	Time to conception (the time from when a woman began trying to conceive until she missed her first period)	Age, body mass index, education, age at menarche, number of previous pregnancies, frequency of sexual intercourse, and number of previous miscarriages	8
Wilsnack (1984)	USA/National samples	Cohort	1981	248730.36	Above 21 years	One year	SAQ/Drinks a day	Any alcohol: None; < 0.22 ounces/day; 0.22–0.99 ounces/day; ≥ 1 ounces/day;	Able to become pregnant after trying at least 1 year	Income, education, smoking.	7
Lopez-del Burgo (2015)	Spain/University graduates from all over Spain	Case-control	1999–2013	686	29.3 (4.2)	Two years	FFQ/Drinks per week	Any alcohol/Wine only: None; ≤ 1/week; 1–5/week; ≥ 5/week; Beer/Spirts only: None; ≤ 1/week; > 1/week;	Difficulty to getting pregnant in the previous year	BMI (4 categories), smoking status (3 categories), leisure-time physical activity (METs-h/week), use of vitamin supplements, adherence to the traditional Mediterranean diet and total energy intake	8
Taylor (2011)	USA/Office workers	Case-control	1990–1994	470	31.03 (20–41)	8 months	SAQ/Drinks per day	Any alcohol: None; < 1 drink/day; ≥ 1 drink/day;	Waiting time to pregnancy (the number of menstrual cycles); the subclinical pregnancies were detected by measuring human chorionic gonadotropin (hCG) levels	BMI, age, smoking status, caffeine, unprotected intercourse during the ovulatory window, trying to get pregnant	8
Greenlee (2003)	Canada/ Agricultural women	Case-control	1997–2001	644	29.7 (18–35)	Two years	SAQ/Drinks per week	Any alcohol: None; 1–2 per week; 3–6 per week; ≥ 7 per week;	Waiting time to pregnancy < 12 months	Education, income, smoking status, number of cigarettes smoked per day, passive smoke exposure, time spent reviewing exposure lists, weight pattern during adult life, male partner’s age, woman’s age at menarche, and number of sexual partners	7
Juhl (2003)	Denmark/ Nationwide women	Case-control	1997–2000	29844	30.36 (25–35)	One year	FFQ/Drink per week	Wine/Beer/Spirits only: None; 0.5–2 per week; 2.5–7 per week; > 7 per week;	Waiting time to pregnancy < 12 months	Age, parity, smoking, BMI, occupational status, and pelvic inflammatory diseases or abdominal diseases	8
Parazzini (1999)	Italy/Randomly selected at the Clinic	Case-control	1990–1995	1769	32 (22–43)	Two years	SAQ/Drinks per day	Any alcohol: None; 1–2 drinks/day; 3 drinks/day;	Difficulty in conception which was defined as taking two or more years to conceive or receiving medical treatment	Age, education, history of spontaneous abortion, and smoking.	8
Jensen (1998)	Denmark/A nationwide trade union members	Case-control	1992–1995	430	25.21 (20–35)	Six months	SAQ/Drinks per week	Any alcohol: None; 1–5 drinks/week; 6–10 drinks/week; 11–15 drinks/week; > 15 drinks/week; Wine/Beer/Spirits only: None; 1–5 drinks/week; > 5 drinks/week;	Obtaining a clinically recognized pregnancy	Women’s cycle number, smoking in either partner and smoking exposure in utero, centre of enrolment, diseases in female reproductive organs, women’s body mass index, use of oral contraception before conception attempt, sperm concentration.	8
Grodstein (1994)	USA/Women enrolled in clinics	Case-control	1981–1983	4023	26.25 (25–34)	One year	SAQ/Gram per week	Any alcohol: None; < 100 g/week; ≥ 100 g/week;	Clinically recognized infertility occurred; infertility was defined as the inability to conceive after 12 months of unprotected intercourse	Age, infertility center, religion, education, body mass index, exercise, cigarette smoking, number of sexual partners, type of contraceptive used, and caffeine intake	8

BMI: Body Mass Index; FFQ: Food-Frequency Questionnaire; NA: Not Available; NOS: Newcastle-Ottawa Scale; SAQ: Self-Administered Questionnaire.

The outcome was defined as waiting time to pregnancy in twelve studies^5,6,8,11,14,15,18,22,23,25–27^, five studies reported infertility occurrence as outcome^9,10,20,21,24^, and two studies reported ovulatory infertility^7,19^. Meanwhile, eleven studies^8–11,15,18,22–24,26,27^ reported that the outcome was confirmed by participants themselves, and eight studies^5–7,14,19–21,25^ reported by the clinically diagnosed in hospital. Five studies^5,9,18,19,24^ reported the overall alcohol consumption as well as consumption of specific types of alcoholic beverage (beer, wine, and spirits). Eight cohort studies^6,10,14,18–21,23^ reported at least three categories of alcohol consumption and were consequently included into the dose-response analysis. Supplementary table 1 showed the results of study quality assessment in detail. All of the studies collected alcohol exposure information by self-reporting questionnaires, including fourteen studies used self-administered questionnaire^5–8,10,11,14,15,18,20,22,23,25,27^ and five studies used food-frequency questionnaire^9,19,21,24,26^, which might lead to exposure bias. Fourteen studies^5,7–11,15,18,19,21,22,24,25,27^ scored 8 points, four studies^6,20,23,26^ scored 7 and only one study^14^ scored 6. The quality scores ranged from 6 to 8 with a median of 7.7 for methodological assessment.

### Meta-analysis results

**Figure 2:**
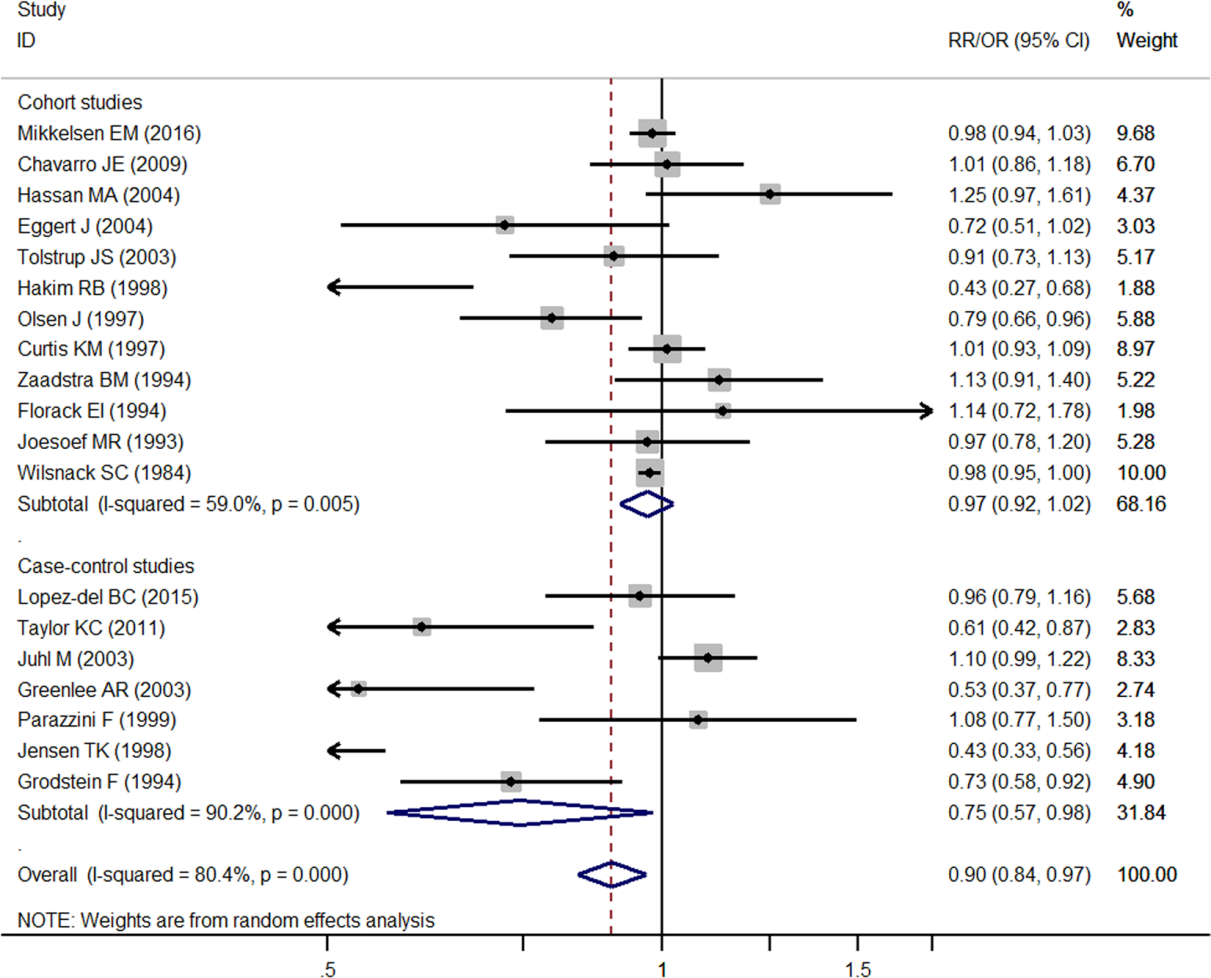
Pooled risk estimates of female alcohol drinking for fecundability (drinkers vs. non-drinkers).

**Figure 3:**
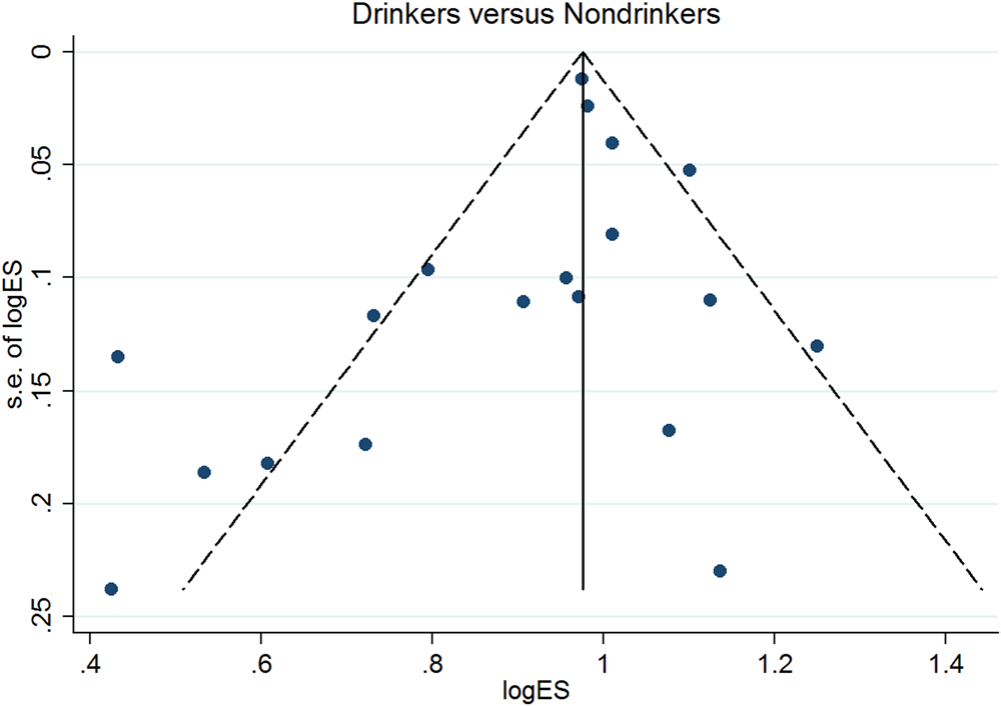
Publication bias by funnel plot.

**Figure 4:**
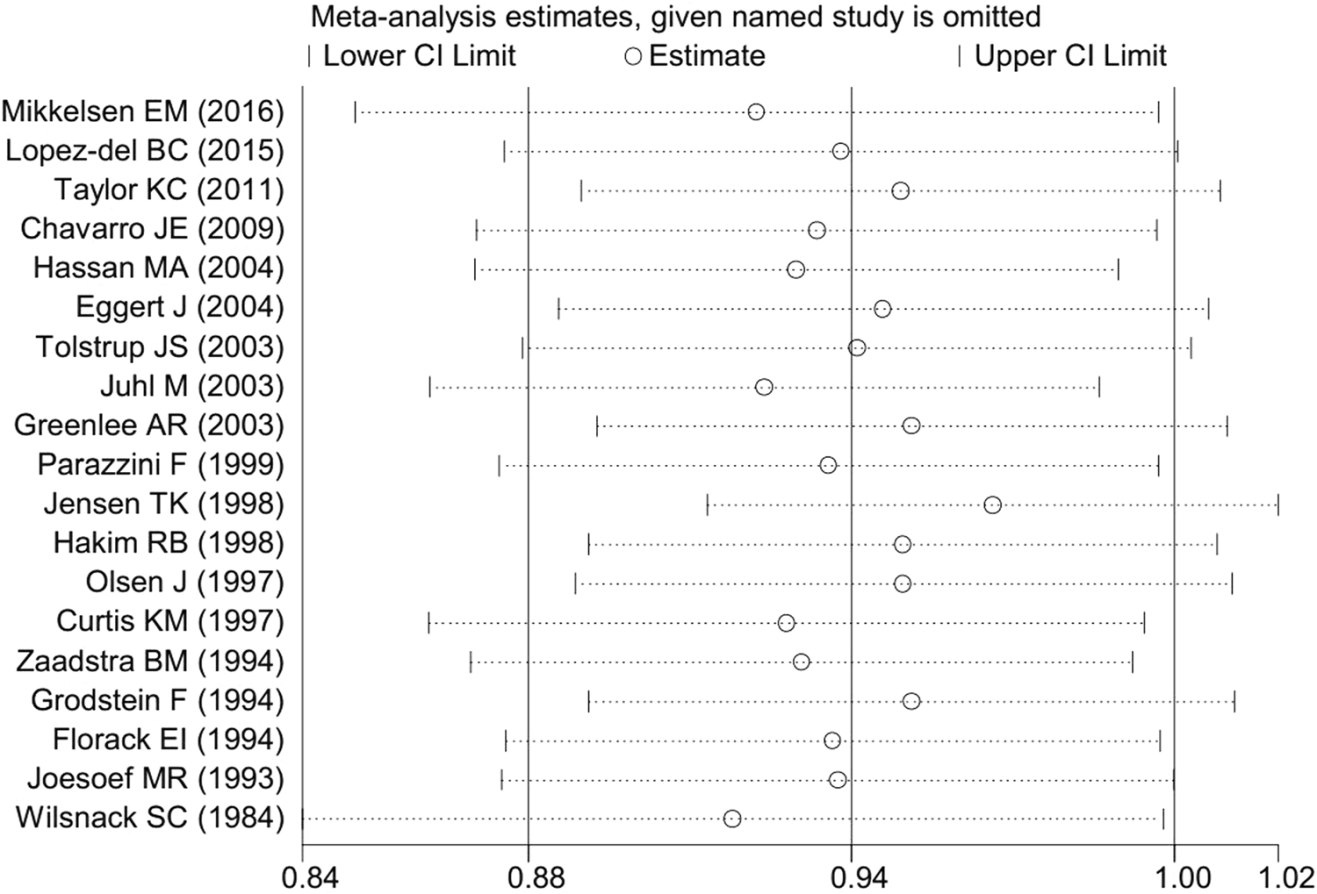
Sensitivity analysis by omitting each study at a time.

Compared to nondrinkers, the combined estimates showed that female alcohol consumption was associated with lower fecundability (0.87 (95% CI 0.78, 0.95)) for overall studies based on 19 studies (*I* ^2^ = 89.6%, *P* = 0.001) (Fig. 2). While the shape of the contour-enhanced funnel plot of studies seemed to be slightly nonsymmetrical (Fig. 3), all the *P* values of Begg’s (*P* = 0.069) and Egger’s (*P* = 0.169) test were more than 0.05 (Table 2), indicating the absence of publication bias. Figure 4 showed the results of sensitivity analysis by omitting each study at a time. No study significantly influenced the overall estimates. The pooled estimates for any drinking varied from 0.92 (when excluding Wilsnack *et al* ^23^) to 0.97 (when excluding Jensen *et al* ^5^).

**Table 2:** Pooled risk estimates of fecundability and female alcohol consumption in subgroup results.

Subgroup analysis	No. of studies	Summary RR (95%CI)	Heterogeneity		Publication bias		P value for Heterogeneity
			*P*	*I* ^2^(%)	Begg’s test	Egger’s test	
Overall	19	0.87 (0.78, 0.95)	0.001	89.6	0.069	0.169	
Dose							
Non vs. lighter	15	0.89 (0.82, 0.97)	0.001	90.3	0.113	0.412	0549
Non vs. moderate	14	0.77 (0.61, 0.94)	0.001	90.7	0.381	0.152	
Study design							
Cohort	12	0.93 (0.86, 1.00)	0.001	77.3	0.732	0.761	0.157
Case-control	7	0.77 (0.53, 1.01)	0.001	92.6	0.230	0.040	
Geographical area							
Europe	11	0.93 (0.78–1.08)	0.001	90.2	0.755	0.644	0.322
America	8	0.80 (0.67–0.93)	0.001	89.8	0.009	0.055	
Type of population							
General	8	0.87 (0.76, 0.98)	0.001	93.1	0.108	0.222	0.854
Hospital	5	1.00 (0.81, 1.20)	0.020	65.8	0.806	0.692	
Worker	3	0.65 (0.35, 0.94)	0.021	74.2	0.999	0.858	
Agricultural	2	0.78 (0.32, 1.25)	0.001	94.1	0.999	—	
Nurses	1	1.01 (0.85, 1.17)	—	—	—	—	
Women’s mean Age							
<25	2	0.84 (0.69, 1.00)	0.273	16.9	0.999	—	0.540
25–30	10	0.87 (0.72, 1.02)	0.001	91.9	0.474	0.315	
≥30	5	0.84 (0.56, 1.12)	0.001	91.6	0.086	0.076	
NA	2	0.98 (0.95, 1.00)	0.970	0	0.999	—	
Time_exposure							
Half-year	4	0.75 (0.42, 1.09)	0.001	95.9	0.308	0.185	0.501
One-year	9	0.95 (0.88, 1.01)	0.002	67.5	0.917	0.796	
Two-years	6	0.84 (0.60, 1.09)	0.001	87.4	0.452	0.127	
Definition of outcome							
Waiting Time to Pregnancy	12	0.85 (0.73, 0.96)	0.001	92.8	0.537	0.364	0.832
Infertility Occurrence	5	0.91 (0.77, 1.06)	0.008	71.0	0.462	0.043	
Ovulatory Infertility	2	0.87 (0.60, 1.15)	0.022	80.9	0.999	—	
Diagnostic method of outcome							
Self-reported	11	0.97 (0.91, 1.03)	0.001	67.5	0.876	0.792	0.043
Clinically-confirmed	8	0.74 (0.55, 0.93)	0.001	88.6	0.035	0.046	
Type alcoholic							
Wine	5	0.98 (0.85, 1.11)	0.001	91.4	0.327	0.264	
Beer	5	1.02 (0.99, 1.05)	0.137	42.7	0.327	0.303	0.242
Spirits	5	0.92 (0.83, 1.01)	0.413	0	0.327	0.977	
Method of alcohol consumption assessment							
SAQ	15	0.83 (0.73, 0.92)	0.001	91.5	0.166	0.945	0.008
FFQ	4	1.03 (0.95, 1.10)	0.295	19.0	0.089	0.002	
Quality score							
NOS = 8	14	0.91 (0.79–1.02)	0.001	88.8	0.324	0.284	0436
NOS ≤ 7	5	0.76 (0.49–1.02)	0.001	92.6	0.086	0.250	

FFQ: Food-Frequency Questionnaire; NA: Not Available; NOS: Newcastle-Ottawa Scale; SAQ: Self-Administered Questionnaire; RR: Risk Ratio.

### Subgroup results

Results of stratified analyses were showed in Table 2 by study design (cohort and case-control) (Fig. 2), geographical area (Europe and America), type of population (general population, hospital clinics, workers, agriculturist, and nurses), women’s mean age (<25, 25–30, and ≥30), the time between alcohol consumption and outcome (half-year, one-year, and two-year), definition of outcome (waiting time to pregnancy, infertility occurrence, and ovulatory infertility), diagnostic method of outcome (self-reported and clinically-confirmed), types of alcoholic beverage (wine, beer, and spirits), method of alcohol consumption assessment (self-administered questionnaire and food-frequency questionnaire), and quality score (NOS = 8 and NOS ≤ 7).

The results implied that female alcohol consumption reduced fecundability in America area (0.80 (95% CI 0.67, 0.93)), general population (0.87 (95% CI 0.76, 0.98)), worker population (0.65 (95% CI 0.35, 0.94)), waiting time to pregnancy as the definition of outcome (0.85 (95% CI 0.73, 0.96)), clinically-confirmed diagnosed method of outcome (0.74 (95% CI 0.55, 0.93)), and self-administered questionnaire method of alcohol consumption assessment (0.83 (95% CI 0.73, 0.92)), respectively. Other subgroup results showed no reduction in fecundability. Most of the results still showed significant heterogeneity in subgroup analyses. Testing by meta-regression method, the heterogeneity could be explained by differences of diagnostic method of outcome and method of alcohol consumption assessment (Table 2).

### Dose-response analysis

Table 2 showed the pooled estimates for the association between light and moderate-heavy drinking and lower fecundability. Compared to nondrinkers, the pooled estimates were 0.89 (95% CI 0.82, 0.97) for light based on fifteen studies (*I* ^2^ = 90.3%, *P* = 0.001) and 0.77 (95% CI 0.61, 0.94) for moderate drinkers based on fourteen studies (*I* ^2^ = 90.7%, *P* = 0.001). The *P* values of Begg’s test were 0.113 and 0.381, respectively, and Egger’s tests were 0.412 and 0.152, respectively (Table 2). These indicated that there was no publication bias for the light and moderate-heavy drinking.

**Figure 5:**
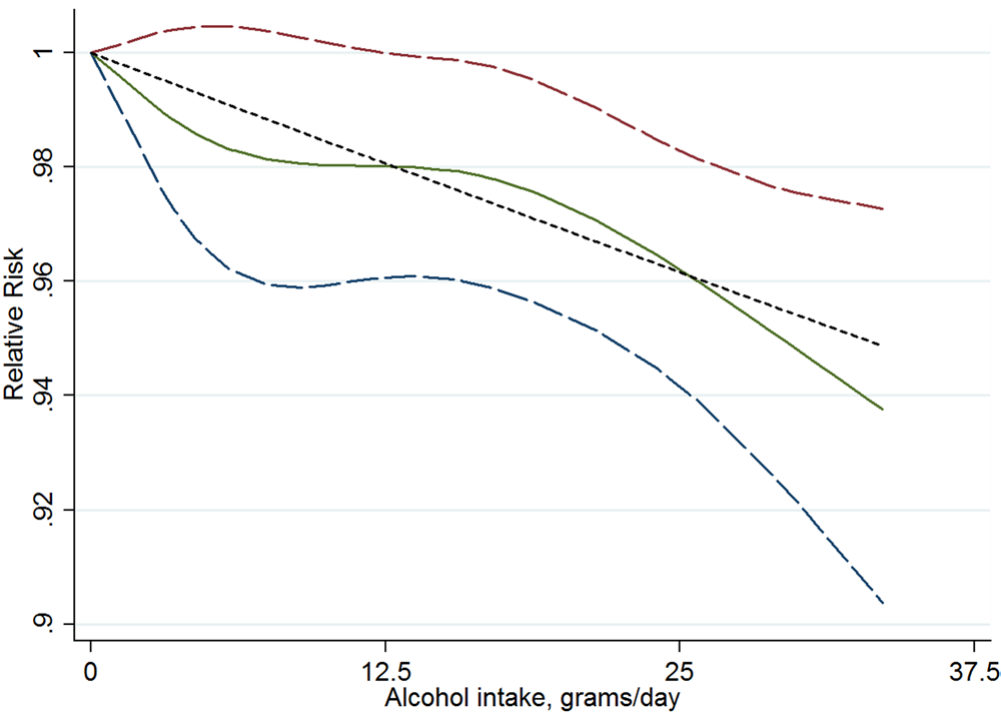
Relative risk (RRs) and the corresponding 95% confidence intervals (CIs) for the dose-response relationship between alcohol drinking (grams per day) and fecundability. The solid line and the long dash line represent the estimated RRs and their 95% CIs. Short dash line represents the linear relationship.

As showed in Fig. 5, no significant difference between the linear line and curve was observed (*P* = 0.119). The dose-response analysis suggested there was evidence of a dose-response relationship between alcohol consumption and decreased fecundability (*P* = 0.001). Dose-response meta-analysis suggested a linear association between decreased fecundability and every 12.5 g/d increasing in alcohol consumption with a RR 0.98, (95% CI 0.97–0.99).

The midpoint was redefined as exposure dose where the lowest category was open ended. The related results were reanalyzed and not substantially altered. The similar results indicated the stability of this meta-analysis (Supplementary Table 2).

## Discussion

This is the first dose-response meta-analysis which aims to investigate the association between female alcohol consumption and fecundability. Using data from 19 studies that involving 98657 reproductive age women, we found that, in relation to nondrinkers, drinking was significantly associated with a 13% (for any drinking), 11% (for light drinking: < 12.5 g/day), and 23% (for moderate-heavy drinking: > 12.5 g/day of ethanol) reduction in fecundability. Importantly, the dose-response analysis showed that women who consumed more than 1 alcoholic drink (12.5 grams of ethanol), will lead to 2% decrease in fecundability. However, there was high heterogeneity in the analysis.

A lot of publications have indicated the association between female alcohol consumption and the fecundability in the past few decades; however, the results were largely controversial^6,8,18,23^. These inconsistencies may be attributed to several factors, including difference in outcome indicators, type of alcoholic beverage consumption^5,9,18,19,24^, sample characteristics, such as lifestyle, age, parity, and study design, such as case-control or cohort study^21,25,26^.

A case-control study among 430 Danish couples aged 20–35 years found that light wine intake, but not beer or spirits intake, was associated with decreased fecundability^5^. By contrast, another study found that wine drinkers have slightly shorter waiting times to pregnancy than both non-wine drinkers and consumers of other alcoholic beverages (beer or spirits)^9^. It is not yet clear why researcher have distinguished between different types of beverages. One explained that wine drinkers generally have healthier lifestyles, fewer infections that unlikely to cause sterility, partners with better sperm quality, more appropriate timing or chances of intercourse^9^. In our subgroup analysis, we found all of the three alcoholic beverages drinking, compared with nondrinkers, were not associated with fecundability. Given the small number of studies (only five), the results need a larger sample to further verify.

High heterogeneity between studies was found in this dose-response meta-analysis. Through stratified and meta-regression analysis, this heterogeneity could be explained by the diagnostic method of outcome (self-reported at home vs. Clinically-confirmed in hospital) and method of alcohol consumption assessment (self-administered questionnaire vs. food-frequency questionnaire).

A lack of objectivity and variability of alcohol consumption may have occurred because information on alcohol exposure history is obtained by self-report in most included studies, and these might have affected the results. Researchers found that participant self-reports could be influenced by deliberate over- or underestimation of alcohol consumption and by failures of memory and other cognitive factors in a clinical trial^28^. To minimize information bias, researchers^7,26^ have suggested that data should be collected by trained interviewers and validated by comparing a subset of verbal responses with information recorded in participants’ medical records in further similar studies.

For alcohol consumption and fecundability, different ethnicities, diagnostic method of outcome and dietary habits could be also explained a part of the disparity in alcohol sensitivity. It has been reported that the distribution of human liver alcohol dehydrogenase (ADH2) and the aldehyde dehydrogenase (ALDH2), which are the principal enzymes responsible for the metabolism of ethanol, differs in different populations^29^. Researchers also found that clinical diagnosis might be an insensitive outcome measure in study of alcohol consumption and infertility^21,30^. Meanwhile, a population-based case-control study from the UK showed that healthy diet might help women in early pregnancy reduce the risk of miscarriage^31^. Similarly, a case-control study nested in a Spanish cohort of university graduates showed a greater adherence to the Mediterranean-type dietary pattern may enhance fertility^32^.

Many observational studies have been published on the topic of dose-response relationship between female alcohol consumption and the effects on the development of fecundability. However, the results on the associations of low to moderate alcohol consumption with fertility showed inconsistent. Results from a prospective cohort study of Danish female residents showed that the frequency of alcohol intake was not associated with adjusted fecundability^18^. In contrast, another prospective study of 7393 healthy women in Sweden found high alcohol consumption was associated with increased risk of infertility^20^. In addition, in a study of 124 women, researchers found that alcohol consumption had an independent dose-related negative effect on the ability to conceive^7^. In this dose-response meta-analysis, we found an inverse association between whole alcohol intake and fecundability. In reproductive age women, each 1 alcoholic drink (12.5 grams of ethanol) increase will decrease the fecundability by 2% (RR = 0.98, 95% CI 0.97, 0.99).

Alcohol consumption has been suggested to affect the age of natural menopause. The data from a recent systematic review and meta-analysis indicated that alcohol consumption, particularly low and moderate alcohol intake, might be associated with later onset of menopause^33^. However, the magnitude of the association is low. Most included women are less than thirty-year-old in this dose-response meta-analysis, and they are still a long way from the onset of natural menopause. Therefore, it was difficult in this study to corroborate the association of alcohol consumption and the onset of menopause.

The biological mechanisms of why which alcohol could impair fertility are still not well clarified. One hypothesis is that alcohol may reduce fecundability through alternating the endogenous hormone concentrations. Previous study has found that 14 drinks a week, compared with no alcohol intake, is associated with increased concentrations of total estrogen, which could reduce FSH secretion suppressing folliculogenisis and ovulation^34^, and the amount of bioavailable estrogen^35^. Another possible cause could be that alcohol has a direct and negative effect on ovum maturation, ovulation, early blastocyst development and implantation^20^. Alcohol intake may be correlated with the intake of other toxicants present in alcoholic beverages, such as ethy1 carbamates, tetra-beta carbolines or food additives, or other substances, such as cooked meat^25^.

This first systemic review and dose-response meta-analysis included many studies with varied populations and a large number of participants in whom the associations between female alcohol consumption and fecundability had been examined. Other strengths of the current study included the quantification of alcohol consumption (grams/day), the enhancement of comparability across studies through the standardization of alcohol consumption, the high quality of included studies, linear and non-linear dose-response analyses, and the detailed subgroup, sensitivity, and influence analyses.

This systemic review and meta-analysis did, like others with similar design, have some potential limitations that should be important to deal with. First, high heterogeneity was detected in the analysis of whole alcohol. Although subgroup analyses and meta-regression were found that diagnostic method of outcome and method of alcohol consumption assessment contributed more or less to the heterogeneity, the source of high heterogeneity was still not found in other potential factors. Second, in consideration of only English publications in four databases were included in this study, these enrolled studies may be not integrated enough as a result of language and database restrictions. In addition, because we are not authorized to use the Embase, the biomedical literatures were not searched in this database. Although Embase and ScienceDirect are both provided by Elsevier, and also, PubMed and Embase can be complement each other in literature searches^36^, potential articles may be also unretrieved. To reduce the effects, manual search was used from the reference list of relevant studies. Meanwhile, it has been found that language restriction did not affect the final result in systematic review^37^. Third, although we took into account the different amounts and ranges of alcohol consumption between studies in the dose-response analysis, studies could also have differed by the types of alcoholic beverage consumed, by how accurately they measured alcohol consumption, or by how they defined alcohol concentration. In addition, most studies collected information by self-reporting questionnaires, which might lead to information bias. Last, because all included studies were observational studies, the possibility that the observed results were affected by confounding cannot be ruled out, although most studies controlled for major confounding factors for fecundability.

In summary, this is the first systematic review and dose-response meta-analysis which has revealed female alcohol consumption was associated with a reduced fecundability. Meanwhile, there was a dose-response relationship between alcohol consumption and decreased fecundability. Our findings may form a foundation for proposing counseling for women of reproductive age, and suggested no alcohol intake for women who are pregnant or may become pregnant. However, because of the high heterogeneity of the current evidence, further rigorous studies with detailed quantification of specific types of alcoholic beverage (beer, wine, and spirits) are needed to find a more precise estimate for female fecundability.

## Methods

### Protocol and registration

This systematic review and meta-analysis was conducted in accordance with the Meta-analysis Of Observational Studies in Epidemiology (MOOSE) guidelines^38^ and the proposal for Preferred Reporting Items for Systematic Reviews and Meta-Analyses (PRISMA)^39^. The study protocol was registered with PROSPERO, the International Prospective Register of Systematic Reviews (CRD42016048417, http://www.crd.york.ac.uk/PROSPERO/display_record.asp?ID=CRD42016048417)^40^.

### Search strategy

We conducted a systematic literature search for potentially relevant case-control and cohort studies, which were published in English, by searching four electronic databases (PubMed, Web of Science, Elsevier Science Direct, and Cochrane Library) from the beginning of indexing to May 2016, and updated up to November 1^st^, 2016, with the following terms: (alcohol OR ethanol OR drinking) AND (fecundability OR infertility OR fecundity OR fertility) AND (cohort OR case-control) (detailed search strategies available in the supplementary). Two authors (DZ Fan and L Liu) independently assessed and identified potentially original articles. The relevant reference list of included articles and previous reviews were also searched manually.

### Inclusion and exclusion criteria

Studies were included if the following inclusion criteria were satisfied: 1) cohort study or case-control study published as original articles; 2) assessed female alcohol consumption as an exposure factor (overall or specific types of alcoholic beverage, such as beer, wine, and spirits) and fecundity as an outcome; 3) provided risk estimates (relative risk, odds ratio, or hazard ratio) with corresponding 95% confidence interval (CI) or standard errors or sufficient information to calculate them. Conference abstracts, reviews, or unpublished reports were not considered for inclusion in the meta-analysis. Following the pre-selection procedures, two authors (DZ Fan and L Liu) independently selected the articles (Fig. 1). Disagreements on eligibility were resolved by discussion. If a study was reported more than once on the same dataset, the one with a more detailed result of alcohol exposure and better control of confounding variables was included in the present analysis.

### Data extraction

Two authors (DZ Fan and W Wang) independently extracted data from each included original article using a standardized data extraction form. Study characteristics recorded from each included study were as follows: surname of the first author, year of publication, study design (cohort or case-control), study country, period of enrollment, type of population (general or special), sample size and number of participants in each category, women’s mean age, time between exposure assessment (alcohol consumption) and outcome, the method used to assess alcohol consumption (food-frequency questionnaire (FFQ) or self-administered questionnaire (SAQ)), types of alcoholic beverage (beer, wine, or spirits), definition of alcohol unit, definition of outcome (waiting time to pregnancy, probability of conception, fertility occurrence, difficulty conceiving, prolonged waiting time, overall infertility or just one type (e.g. ovulatory infertility)), diagnostic method of outcome (self-reported at home or clinically-diagnosed in hospital), confounding factors controlled by matching or adjustment, and risk estimates with corresponding confidence intervals. The standardized data extraction form was provided as a supplementary table 3. Where disagreements existed, both authors reviewed the materials together until a consensus was reached.

### Quality assessment

Two authors (DZ Fan and Q Xia) independently assessed the quality of included studies according to the 9-star Newcastle-Ottawa Scale (NOS)^41^, which is a validated scale for observational and non-randomized studies in meta-analysis. The NOS includes three broad perspectives: the selection of the study sample (maximum of four points), the comparability of the sample groups (maximum of two points) and the exposure/outcome (maximum of three points). A maximum quality score was 9 points, and study with awarded points ≥7 was defined as high quality. Disagreements were discussed and resolved by consensus.

### Statistical analyses

The presentation of the quantity of alcohol consumption varies among different studies. In preparation for the meta-analysis, standardized alcohol consumption was transformed to total grams of ethanol per day. The midpoint of each category was taken as corresponding exposure dose when a series of categories of alcohol intake were given. Of the enrolled studies, where the lowest category was open ended, zero was defined as the lowest exposure dose, and where an upper open-end category was given, 1.2 times its lower bound was used as the exposure dose^42^. From the information in each included studies, they mainly divide into two alcohol units. One is gram per day or week^6,9,10,19–21^ and the other is drinks per week^5,7,8,11,14,15,18,22–27^. For estimation of alcohol consumption, when studies reported alcohol consumption in gram per day or week, we direct convert gram per day; when studies reported in drinks per week, we assumed that one drink contain 12.5 g of alcohol and converted it into g/day, as proposed by previous meta-analysis^43^. For specific types of alcohol beverage, such as beer, wine, spirits, and whisky, when studies reported detailed information, we direct convert gram per day; otherwise, we assumed as above method.

We treated the nondrinkers group as reference category in the meta-analysis. As higher alcohol exposure was labeled more than one drink per day in the majority of the included studies, the alcohol drinkers were divided into two levels: light drinker was defined as ≤1 drink/day (≤12.5 g/day of ethanol) and moderate-heavy drinker as >1 drinks/day (>12.5 g/day of ethanol), as based on similar study^43^. Fecundability was seen as the final outcome in this meta-analysis. When fecundability was not directly reported, it would be re-calculated according to the given data. When the numbers of infertility and total participants in each category were available, risk estimates were then directly re-calculated. If the risk estimates were directly available in the infertility research study, the reciprocal was re-calculated and considered as outcome in each category. Where a study presented a dose-response analysis only, the corresponding risk estimates for all drinking categories were re-calculated based on the method proposed by Hamling *et al* ^44^ when possible. The method was also used for light and moderate-heavy drinker when more than one exposure categories fell in one of these levels.

Statistical heterogeneity among articles was quantitatively assessed using both Q test and *I* ^2^ statistic^45^. A *P* value less than 0.1 in Q-test or a value more than 50% in *I* ^2^ statistic was defined as significant heterogeneity^46^. As a result, a random-effects model would be used to assign the weight of each study according to the DerSimonian-Laird method^47^; otherwise, fixed-effects model would be used. Subgroup analyses in terms of study design, geographic area, type of population, women’s mean age, time between exposure assessment (alcohol consumption) and outcome, method of alcohol exposure assessment, method of outcome definition, types of alcoholic beverage (beer, wine, or spirits), and the quality score were conducted to explore the potential sources of heterogeneity among studies. Furthermore, random-effects meta-regression was also used to assess of heterogeneity^48,49^. As it may different to have a null alcohol consumption than a low alcohol consumption, the midpoint was redefined as exposure dose where the lowest category was open ended. Besides, the related results were also reanalyzed in that case. Sensitivity analyses were also performed to evaluate robustness and stability by excluding each study at a time to clarify the influence of each study on the overall estimates. Publication bias was assessed by the contour-enhanced funnel plot^50^, the Egger regression asymmetry test^51^ and the Begg’s rank correlation test^52^.

Furthermore, a potential dose-response relationship between alcohol exposure and fecundability were conducted, based on the natural logarithm of the RR for each cohort study with at least three quantitative categories of exposure using the methods described by Greenland and Orisini^53,54^. Restricted cubic splines with four knots at percentiles 5%, 35%, 65% and 95% of the distribution were used to evaluate a potential curve association between alcohol exposure and fecundability. The *P* value for curve fitting with linear or nonlinear was calculated by testing the null hypothesis with which the coefficient of the second spline equals to zero.

A two-tailed *P* value less than 0.05 was considered statistically significant, except where otherwise specified. All analyses were performed using Stata 12.0 (Stata Corporation, College Station, TX). Meta-analysis, publication bias and sensitivity analyses were used metan, metabias and metaninf function, respectively.

## Electronic supplementary material

Supplementary Information
